# An emerging role of regulatory T-cells in cardiovascular repair and regeneration

**DOI:** 10.7150/thno.47118

**Published:** 2020-07-11

**Authors:** Tiffany H.W. Fung, Kevin Y. Yang, Kathy O. Lui

**Affiliations:** 1 Faculty of Medicine, Prince of Wales Hospital, The Chinese University of Hong Kong, Hong Kong, China.; 2 Department of Chemical Pathology, Prince of Wales Hospital, The Chinese University of Hong Kong, Hong Kong, China.; 3 Li Ka Shing Institute of Health Sciences, Prince of Wales Hospital, The Chinese University of Hong Kong, Hong Kong, China.

## Abstract

Accumulating evidence has demonstrated that immune cells play an important role in the regulation of tissue repair and regeneration. After injury, danger signals released by the damaged tissue trigger the initial pro-inflammatory phase essential for removing pathogens or cellular debris that is later replaced by the anti-inflammatory phase responsible for tissue healing. On the other hand, impaired immune regulation can lead to excessive scarring and fibrosis that could be detrimental for the restoration of organ function. Regulatory T-cells (Treg) have been revealed as the master regulator of the immune system that have both the immune and regenerative functions. In this review, we will summarize their immune role in the induction and maintenance of self-tolerance; as well as their regenerative role in directing tissue specific response for repair and regeneration. The latter is clearly demonstrated when Treg enhance the differentiation of stem or progenitor cells such as satellite cells to replace the damaged skeletal muscle, as well as the proliferation of parenchymal cells including neonatal cardiomyocytes for functional regeneration. Moreover, we will also discuss the reparative and regenerative role of Treg with a particular focus on blood vessels and cardiac tissues. Last but not least, we will describe the ongoing clinical trials with Treg in the treatment of autoimmune diseases that could give clinically relevant insights into the development of Treg therapy targeting tissue repair and regeneration.

## Introduction

As the healthcare of the population improves, the life expectancy of people has been increasing. However, a significant proportion of the population is crippled by chronic non-communicable diseases, placing huge burdens on the societies of the developed countries. These chronic conditions, including the most common co-morbidities: type 2 diabetes, Alzheimer's disease, hypertension, coronary artery disease, and heart failure; are associated with the dysregulation of immune system leading to chronic inflammation 1-5. Currently the therapeutic approach focuses on the prevention of progression and deterioration for these diseases, but there is no complete cure. Therefore, extensive research has been performed in the search for alternative treatment options.

The immune system has been a particular field of interest, as it can regulate not only the physiological processes of inflammation, but also tissue repair and regeneration. However, excessive anti-inflammatory response in the resolution process could lead to pathological fibrosis. Therefore, a tight regulation of the anti-inflammatory response dictates the outcome whether the inflamed tissue is replaced by scar tissue, or replaced with the regenerated tissue to restore organ function after injury 6. The study of the immune cell mediators essential to the repair and regeneration of injured tissue is attractive to search for new therapeutic targets, although the mechanisms by which the immune cells coordinate to mediate organ repair and regeneration have been less studied.

Regulatory T-cells (Treg) are well-known for their immunomodulatory properties to resolve inflammation or prevent the inappropriate activation of inflammation leading to autoimmunity. Apart from this, there is an emerging evidence that they participate in local tissue regeneration, and by manipulating such property of Treg to tip the balance from fibrotic resolution that usually occurs in adults, to a pro-regenerative one with restoration of organ function. This review is a compilation of the overall picture of the spectrum of Treg function regarding their immunomodulatory and reparative functions, with specific focus on the most recent findings on the role of Treg in angiogenesis and cardiomyocyte regeneration.

## The Immune Role of Treg in Tolerance

**Figure 1:**
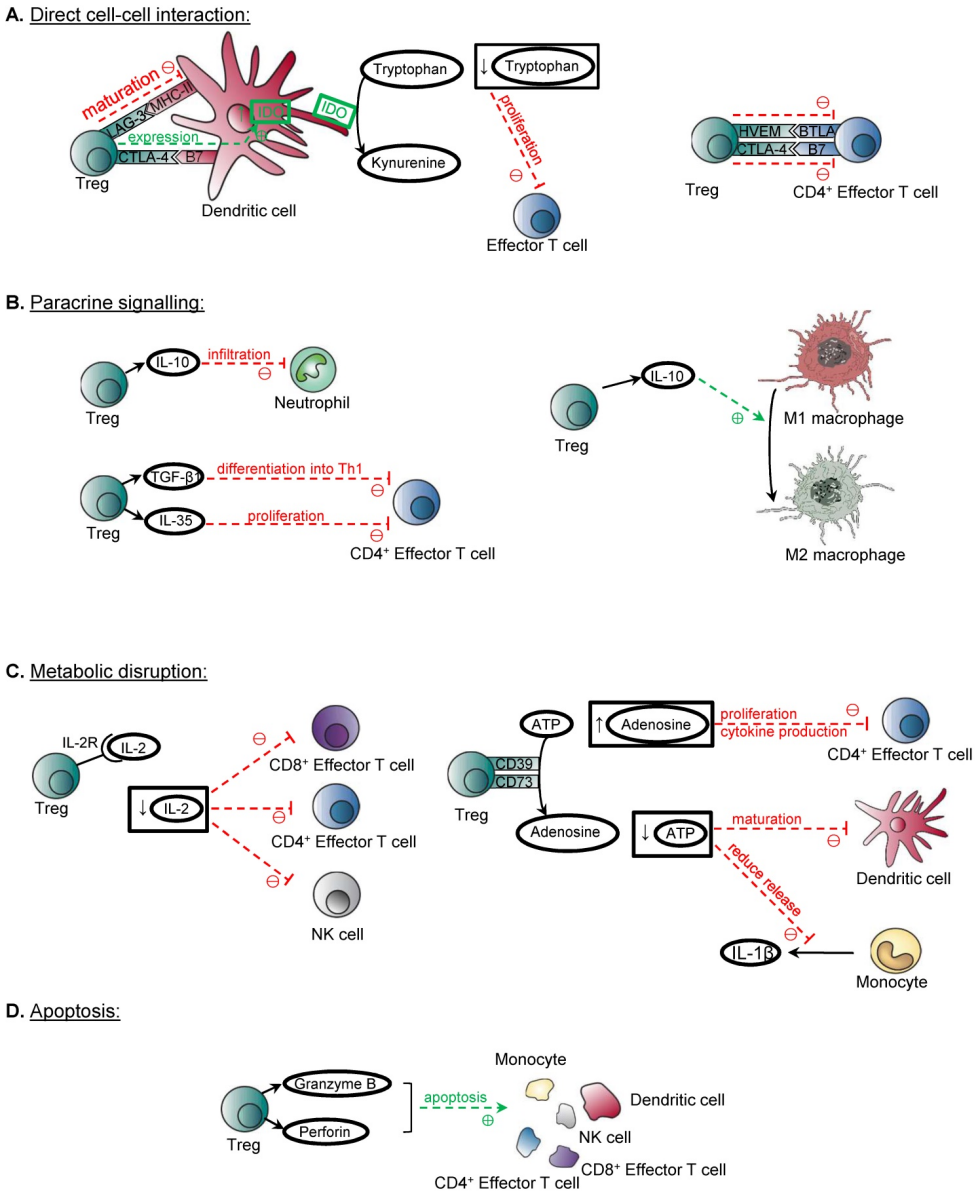
** Mechanisms of immune suppression by Treg.** (**A**) Treg can suppress their target cells through direct cell-cell interaction. CTLA4/B7 interaction between Treg and dendritic cells can induce IDO production by dendritic cells, and the overall effect of reduction in tryptophan suppresses the proliferation of effector T cells. LAG3/MHCII interaction between Treg and dendritic cells suppresses dendritic cell maturation. CD4^+^ effector T cells are suppressed by the HVEM/BTLA and CTLA4/B7 interaction with Treg. (**B**) Treg also demonstrates paracrine signalling to suppress its targets. IL-10 is shown to suppress the infiltration of neutrophils in the kidney after ischaemic-reperfusion injury. IL-10 can also induce the conversion from M1 macrophages to M2 macrophages. TGF-β1 is also demonstrated to suppress the differentiation of Th1 cells and prevent the development of colitis. IL-35 can suppress the proliferation of CD4^+^ effector T cells. (**C**) Another mechanism is metabolic disruption. High affinity IL-2 receptors expressed by Treg depletes IL-2, and this suppresses the activation of CD8^+^ T cells, CD4^+^ T cells and NK cells. The ectoenzymes CD39 and CD73 expressed on Treg cell surface catalyse the conversion of ATP to adenosine. Increased adenosine levels inhibit the proliferation and cytokine release by activated CD4^+^ effector T cells. The overall effect of reduction of ATP leads to reduced maturation of dendritic cells and reduced release of pro-inflammatory IL-1β by monocytes. (**D**) Treg can induce apoptosis on immune mediators through granzyme/ perforin pathway, and they include monocytes, dendritic cells, NK cells, CD4^+^ and CD8^+^ T cells.

Treg are considered as key mediators for the establishment of peripheral tolerance, as their immunosuppressive function dampens or prevents the immune system to mount an inflammatory response inappropriately towards self-antigens, commensal microbiota, food and environmental antigens, which would otherwise lead to the deleterious effects of autoimmunity and allergy, respectively 7, 8. Treg have also been shown to play a pivotal role in preventing the rejection of murine and human stem cell transplants 9, 10. The development of Treg from the thymus and periphery has been reviewed extensively 11, 12. Several mechanisms have been proposed to be deployed by Treg to exert their immunosuppressive function on the effector cells of both the innate and adaptive arms of immunity, including macrophages, neutrophils, NK cells, dendritic cells, and T cells particularly CD4^+^ and CD8^+^ T cells. The mechanisms of action include direct cell-cell interaction, paracrine signalling, metabolic disruption and induction of apoptosis (Figure 1).

The molecules expressed on the surface of Treg which have been identified to confer their suppressive functions include CTLA-4, LAG-3, and HVEM. For instance, Treg can suppress dendritic cells through CTLA-4, which binds with B7 molecules CD80 and CD86 on dendritic cells to induce the production of indoleamine 2,3-dioxygenase (IDO) by dendritic cells that suppresses the proliferation of effector T cells 13, 14. LAG-3 expressed on Treg can also inhibit the maturation of dendritic cells through binding to their MHC-II 15. Moreover, Treg can directly suppress CD4^+^ effector T cells through the binding of the inhibitory costimulatory molecules HVEM/BTLA 16, and CTLA4/B7 17.

The release of cytokines IL-10, TGF-β, and IL-35 is crucial to the immunosuppressive functions of Treg, as shown in their regulation of allergic inflammation including asthma, and other autoimmune diseases 18-20. These cytokines are able to directly suppress or pose the polarization of a pro- to anti-inflammatory phenotype of the immune cells including neutrophils, macrophages and effector T cells. For example, the ability of Treg to suppress the infiltration of neutrophils in the kidney after ischaemic-reperfusion injury is IL-10 dependent, as this phenomenon is abolished in cell specific knockout of IL-10 in Treg 21. Treg can also target the pro-inflammatory M1 macrophages and the release of IL-10 is thought to contribute to the induction of their conversion to anti-inflammatory and pro-repair M2 macrophages 22. Despite the controversy surrounding whether the expression of TGF-β is essential for Treg suppressive function, more recent evidence from transfer models of TGF-β1-deficient Treg shows that TGF-β1 produced by Treg is important in the prevention of colitis by suppressing the differentiation of Th1 cells 23 that are the mediators of the development of colitis 24. IL-35 has also been identified to play an important role, as recombinant IL-35 alone was demonstrated to suppress the proliferation of CD4^+^ effector T cells *in vitro* 20.

Furthermore, Treg have been demonstrated to regulate immune cell mediators through metabolic disruption. Whether competitive consumption of IL-2 with effector T cells through the high affinity IL-2 receptors on Treg has been a controversial topic, but more recent evidence has shown that interruption of the IL-2 signalling pathway is an essential mechanism in suppressing the activation of CD8^+^ T cells, whereas IL-2 is dispensable for the control of CD4^+^ T cells 25. Depletion of IL-2 has also been shown to suppress the activation of NK cells *in vivo*, suggesting that IL-2 is essential for the activation of both the innate and adaptive arms of immunity 26. Another mechanism of suppression by Treg involves the production of adenosine. Treg express ectoenzymes CD39 and CD73 on the surface, which catalyse the degradation of nucleotides such as ATP into adenosine. The overall effect is an anti-inflammatory one, as adenosine can inhibit the proliferation and production of cytokines by activated CD4^+^ effector T cells through binding to A2A receptors, whereas the depletion of ATP, a pro-inflammatory signal, implies that there is reduced ATP-induced dendritic cell maturation, or ATP-induced release of pro-inflammatory cytokine IL-1β by monocytes 27-29.

Last but not least, Treg demonstrate the ability to induce apoptosis of immune cell mediators, through the granzyme/ perforin pathway. Evidence has shown that Treg can release Granzyme B, and elicit apoptosis of target cells including monocytes, dendritic cells, NK cells, CD4^+^ and CD8^+^ T cells through a perforin-dependent pathway 30, 31. Moreover, it is suggested that Treg can also exert the cytotoxic effects through the TRAIL/DR5 pathway, as the inhibition of this pathway abolishes the cytotoxic and suppressive effects of Treg on CD4^+^ T cells* in vitro* 32.

## The Tissue Diversity of Treg

Previously, Treg are divided into two major groups according to their origin, namely thymus-derived (tTreg), and peripherally-derived (pTreg) Treg. The latter is a group of Treg formed by conversion of conventional CD4^+^ T cells in the periphery. However, this classification is not sufficient to represent the diversity and heterogeneity of Treg, as further characterisation of the phenotype and function of Treg shows that distinct subpopulations of Treg reside in non-lymphoid tissues, and display non-immune functions that are unique to the tissue type. Each tissue-resident Treg population is unique in terms of the expression of transcription factors, recruitment by chemokines and adhesion receptors, and effector functions 33.

Treg that reside in the adipose tissue are the best-characterised tissue-resident Treg so far. They comprise more than half of the CD4^+^ T cell population, compared to those found in lymphoid organs that contain less than 30% of the CD4^+^ T cell population 34. At the gene expression level, compared to those residing in the lymphoid tissue, adipose tissue-resident Treg upregulate genes that are involved in leukocyte migration and extravasation (Gm1960, CCR1, CCR2, CCR9, CCL6, integrin alpha V, Alcam, CXCL2, CXCL10), and, in particular, the *Il10* transcript is overexpressed by 136-fold compared to those of the lymphoid tissue 34. PPAR-γ is also over-expressed in adipose tissue-resident Treg that is identified as the factor important for their accumulation, phenotype and function in adipose tissue 35. The function of adipose tissue-resident Treg has been suggested to suppress the inflammation in adipose tissue and to maintain insulin sensitivity, as the depletion of adipose tissue-resident Treg to 30% of the usual levels shows increase in inflammatory mediators in visceral fat depot and insulin resistance 34.

Tissue-resident Treg are unique in their phenotype and function, as Treg that reside in other tissues such as the skin have distinctly different phenotype and function compared to that of the adipose tissue-resident Treg. Treg that reside in the skin are characterised by the expression of CCR4 and CD103 36. The role of skin-resident Treg is to suppress autoimmune and overactive inflammatory responses, as shown in experiments with the transfer of Fut7-deficient Treg to neonatal scurfy mice. Scurfy mice lack Treg, and Fut7 is an enzyme that produces E- and P-selectin, which are required for homing of Treg to the skin. Transfer of Fut7-deficient Treg to neonatal scurfy mice induced dermatitis, which is not seen after transfer of WT Treg 37. Consistent with the results in mice, human patients with autoimmune skin conditions including systemic sclerosis and dermatomyositis show reduced Treg levels and reduced serum levels of TGF-β and IL-10 38, 39.

Altogether, adipose tissue- and skin-resident Treg are examples illustrating the diversity in the phenotype and function of tissue-resident Treg. In fact, they have been identified in many non-lymphoid organs such as the lung, liver, pancreas, skeletal muscle, and intestine 40. Whether these resident Treg represent a homogenous population of a particular tissue type remains elusive. Future studies should focus on the cellular, molecular and functional heterogeneity of resident Treg of each tissue type at a single cell resolution.

## The Reparative Role of Tissue-Resident Treg

**Figure 2:**
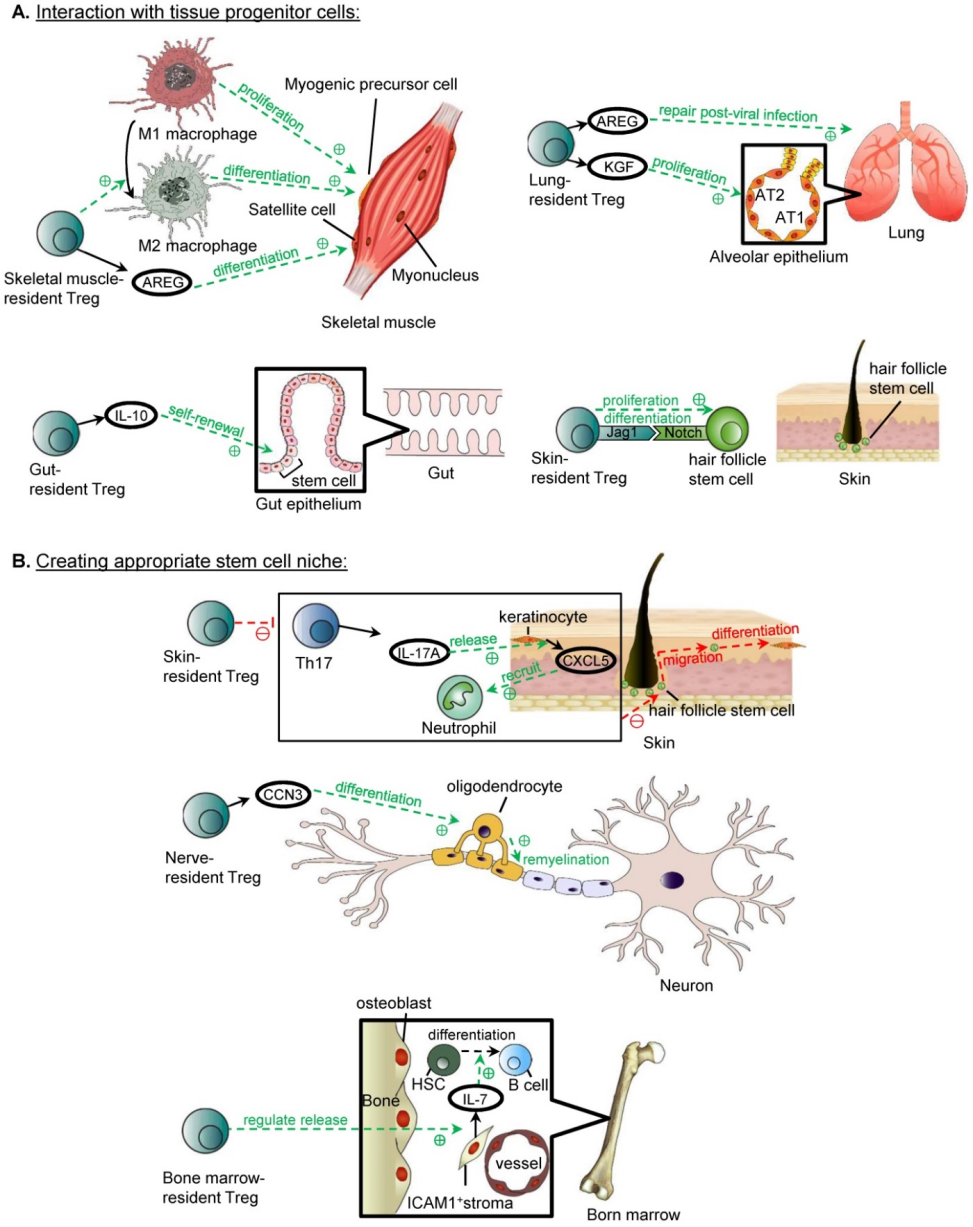
** Reparative role of tissue-resident Treg.** (**A**) Treg can promote repair through direct interaction with tissue progenitor cells. Skeletal muscle-resident Treg promotes the conversion of M1 to M2 macrophages, and the timely switch is important in muscle repair, as M1 macrophages are responsible for inducing proliferation of myogenic precursor cells, whereas M2 macrophages promote the differentiation of myogenic precursor cells. Skeletal muscle-resident Treg also releases AREG which stimulates the differentiation of satellite cells. Lung-resident Treg releases AREG and KGF, and AREG is important lung tissue repair post-influenza infection, whereas KGF induces the proliferation of AT2 cells in the alveolar epithelium. Gut-resident Treg releases IL-10 promotes the self-renewal of stem cells in the intestinal epithelium. Skin-resident Treg in hair follicles can induce the proliferation and differentiation of hair follicle stem cells through Jag1/Notch interaction. (**B**) Treg interact with supportive cells to create an appropriate stem cell niche for tissue repair. Skin-resident Treg inhibit the CXCL5/Th17/neutrophil axis, which includes Th17 releasing IL-17A, IL-17A inducing the release of CXCL5 by keratinocyte, and CXCL5 recruiting neutrophils. The overall effect of the inhibition of the axis is the migration of hair follicle stem cells to the upper epidermis and their differentiation into keratinised epithelial cells for repair of the skin barrier (Adapted with permission from 50, copyright 2019 Elsevier Inc.). Nerve-resident Treg releases CCN3, which is thought to promote oligodendrocyte differentiation, and thus the remyelination of neurones in the central nervous system. Bone marrow-resident Treg regulates the release of IL-7 from ICAM1^+^ perivascular stromal cells, which supports the development of lymphoid lineage including the differentiation of B cells from haematopoietic stem cells (Adapted with permission from 52, copyright 2017 Nature Research).

Tissue-resident Treg exhibit a diverse spectrum of functions, and in fact, Treg residing in some tissue types have displayed reparative functions, including induction of the regeneration of parenchymal cells in skeletal muscle, lung, gut and hair follicle of the skin (Figure 2). They promote tissue repair and regeneration through a direct interaction with the progenitor or stem cells; and/or an indirect effort to build a reparative niche to support the progenitor or stem cells.

### Promoting repair through interacting with tissue progenitor cells

In the skeletal muscle, one aspect of Treg promoting repair and reducing damage from inflammation is through the conversion of macrophages from the pro-inflammatory M1 to anti-inflammatory and pro-repair M2 phenotype. As demonstrated in a model for Duchenne muscular dystrophy, the depletion of Treg resulted in an increased level of activated M1 macrophages, reduced M2 macrophages, and deterioration of muscle damage and inflammation, suggesting that Treg reduce damage and promote repair through regulating the ratio of M1:M2 macrophages 41. The mechanism by which Treg regulate macrophage polarization in the context of tissue repair and regeneration has not been fully studied. One potential mechanism is through suppression of IFNγ produced by CD4^+^ effector T cells, and IFNγ is a promoter of formation of M1 macrophages, but whether there are other direct mechanisms, perhaps related to the increased in IL-10 level in muscle, has not yet been explored or investigated 41. The polarisation of macrophages from a M1 to M2 phenotype is important in muscle repair, as immediately after injury, M1 macrophages take part in the phagocytosis of necrotic debris and induce proliferation of myogenic precursor cells, and the timely switch to M2 macrophages functions to induce the differentiation of myogenic precursor cells for myogenesis 42. A more direct mechanism by which Treg promotes the regeneration of skeletal muscle is mediated through the release of amphiregulin (AREG) that has shown to stimulate the differentiation of satellite cells 43. Satellite cells are activated after muscle injury, and are unipotent adult stem cells that are the source of regenerating muscle fibres 44. Skeletal muscle resident Treg release AREG *in vitro* and *in vivo*, and at significantly higher levels compared to those of the lymphoid organs 43. Treg ablation disrupts the myogenesis from satellite cells post-injury resulting in disorganised muscle fibres and muscle inflammation 43.

The release of AREG by tissue resident Treg is not exclusive to skeletal muscle, and is also demonstrated in the lung. Two mediators released by Treg are implicated in the repair of lung tissue after acute lung injury, including AREG and keratinocyte growth factor (KGF). The release of AREG by lung-resident Treg at as early as day 3 after influenza viral infection, and mice with AREG-deficient Treg display more severe lung tissue injury and deterioration of lung function, suggesting that AREG is important in the repair of lung tissue post-injury and restoration of lung function, although the mechanism underlying how AREG repairs the lung tissue remains elusive 45. Apart from AREG, there is also evidence that lung-resident Treg release KGF after acute lung injury, and KGF is able to induce the proliferation of alveolar epithelium as evidenced by the fact that mice with KGF-deficient Treg have significantly reduced proliferation of alveolar epithelial cells and among these AT2 cells (type 2 alveolar epithelial cell) in particular *in vivo,* and co-culture of KGF-deficient Treg with AT2 cells *in vitro* reduced their proliferation rate 46.

In addition to growth factors, cytokines such as IL-10 released by gut-resident Treg are also important in the maintenance of the stem cell pool by promoting their self-renewal in the intestine, as Treg ablation resulted in an altered differentiation of the intestinal epithelial cells, due to the decreased pool of intestinal stem cells, and the increased accumulation of Th1, Th2, and Th17 cells, where these cells suppress the renewal of intestinal stem cells, and cytokines released by Th1 and Th2 cells can also modulate lineage commitment of these intestinal stem cells, although it has not been shown whether the Treg play a role in regulating the ratio of Th1 and Th2 cells to control the proportion of the differentiated cells 47.

Apart from the release of growth factors by Treg to induce repair, they can also induce the reparative process through contact-dependent mechanisms to activate the progenitor cells directly. In hair follicles, skin-resident Treg mainly reside in the hair follicles 48, and due to the close proximity to the hair follicle stem cells, they can induce regenerative process by the expression of Jag1, which activates the Notch signalling pathway of hair follicle stem cells directly, resulting in their proliferation and differentiation 49.

### Promoting repair through creating an appropriate stem cell niche

In addition to directly working with the stem or progenitor cells, Treg also facilitate repair of local tissue through interacting with the supportive cells of the microenvironment that support stem or progenitor cells in the skin, neural tissue, and bone marrow. In the repair of the epidermal layer of the skin after injury, the migration of hair follicle stem cells to the upper epidermis and their differentiation into keratinised epithelial cells as an alternative fate is required for restoring the skin barrier, and Treg are essential regulators of this process 50. It is thought that one mechanism is mediated by the suppression of the CXCL5/Th17/neutrophil axis, including the inhibition of the release of IL-17A by Th17 cells, the release of CXCL5 by keratinocyte, and neutrophil recruitment as CXCL5 is a neutrophil chemoattractant 50.

Furthermore, Treg have been shown to promote oligodendrocyte differentiation, which is an essential process during the remyelination of neurones of the central nervous system 51. It is hypothesised that this process is mediated by the production of CCN3 by Treg from *in vitro* studies 51. Treg can also maintain the function of bone marrow stromal cells, in particular, and regulate the release of IL-7 from ICAM1^+^ perivascular stromal cells which is important in supporting the development of lymphoid lineage, including the differentiation of B cells from haematopoietic stem cells 52.

The mechanism underlying this requires further investigation, as it is unknown whether the bone marrow-resident Treg interact with the stromal cells directly, or through the suppression of activated CD4^+^ and CD8^+^ T cells, as increased levels of activated CD4^+^ and CD8^+^ T cells are detected in the bone marrow after Treg depletion 52. Nevertheless, follow up study is lacking to determine whether activated CD4^+^ and CD8^+^ T cells alone can negatively regulate the IL-7 production by ICAM1^+^ perivascular stromal cells 52.

## Treg in Cardiovascular Repair and Regeneration

Given that cardiovascular disease is the leading cause of deaths worldwide, novel insights into the development of potential therapeutics against cardiovascular diseases are needed. Recently, accumulating evidence supports the role of Treg in promoting angiogenesis, and facilitating the repair and regeneration of endothelial cells as well as cardiomyocytes. We will focus on the unappreciated role of Treg in the repair of blood vessels and heart muscle in this section.

### Angiogenesis

The first evidence that Treg may play a role in angiogenesis is based from studies in tumours, where tumours exploit such mechanisms to fuel their proliferation. In murine ovarian cancer cell lines, recruited Treg are one of the main sources of VEGF_A_ in the tumour microenvironment, as the levels of VEGF_A_ and tumour microvascular density are significantly reduced after the administration of the anti-CD25 antibody that depletes Treg 53. In addition, hypoxic conditions cause Treg to secrete significantly higher amount of VEGF_A_, and the culture medium of hypoxic Treg is able to induce the expansion of CD31^+^ endothelial cells *in vitro,* leading to significantly greater capillary endothelial network, suggesting an important role for Treg in tumour angiogenesis 53*.*

The findings in hypoxic conditions are consistent with observations in the model of hindlimb ischemia that can recapitulate human peripheral arterial disease. After ligation of the femoral artery, Treg are likely key contributors for the reperfusion of the ischaemic limb, as administration of the anti-CD25 antibody profoundly reduces the extent of reperfusion achieved in the ischaemic limb compared to the control group 54. Similarly, in another study, adoptive transfer of Treg improves the reperfusion of the ischaemic limb compared to the untreated controls; and immunohistochemistry on the ischemic tissues also shows that there are more CD31^+^SMA^+^ blood vessels, suggesting that Treg promote revascularisation of the ischaemic limb *in vivo* 55. Mechanistically, IL-10 has been proposed to be the mediator by which Treg induce post ischemic angiogenesis, as IL-10 mRNA expression is reduced in the ischaemic limb after ablation of Treg by the anti-CD25 antibody; and the administration of IL-10 blocking antibody can completely abolish the beneficial effects from the adoptive transfer of Treg on post ischemic angiogenesis 55.

In addition to VEGF_A_ and IL-10, other Treg associated paracrine factors have been found effective in promoting angiogenesis even in hyperglycaemic conditions. For instance, co-culturing of murine endothelial cells with Treg or Treg culture medium shows increased tube formation, and although the components of the culture medium is not analysed, IL-10 and AREG are shown to be secreted by Treg in other context including in immunosuppressive mechanisms and in repair of skeletal muscle, and culturing the endothelial cells with IL-10 or AREG alone can reproduce the results of increased tube formation, and increase the expression of Apelin that is associated with sprouting angiogenesis, suggesting that these factors could be the underlying mediators 54. Further investigation reveals that Treg or IL-10 can promote the proliferation of endothelial cells directly, which can account for the increased tube formation by endothelial cells, although AREG could act through a different mechanism other than inducing the endothelial cell proliferation to promote angiogenesis 54.

**Figure 3:**
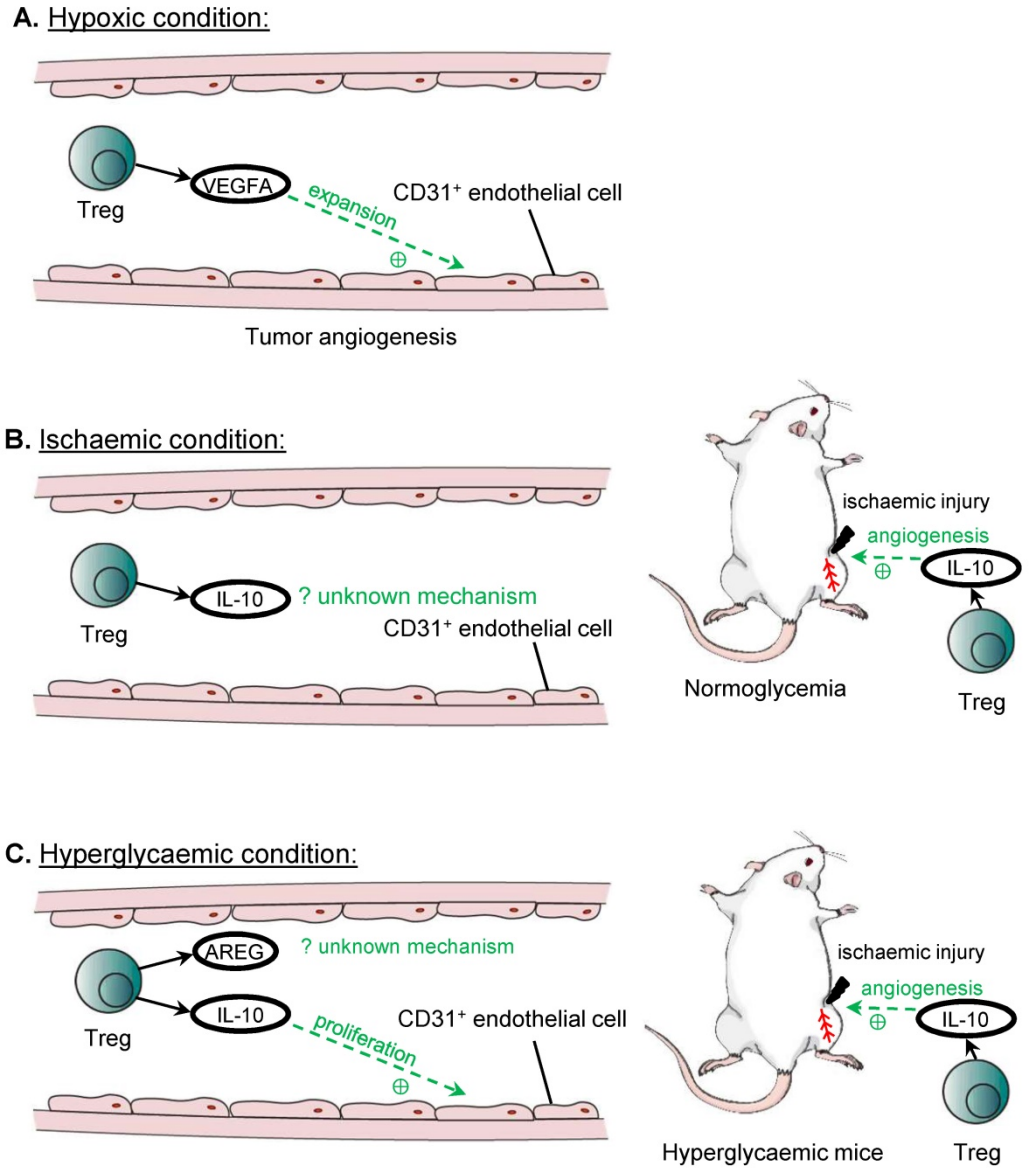
** Treg in angiogenesis.** Treg can promote angiogenesis under hypoxic or hyperglycaemic conditions. The main source of VEGFA for tumour angiogenesis in ovarian cancer cell lines is from recruited Treg, and VEGFA promotes the expansion of CD31^+^ endothelial cells. Treg also promotes angiogenesis in ischaemic hindlimb through the release of IL-10, although the mechanism remains unknown. IL-10 released by Treg also improves hindlimb ischaemia in hyperglycaemia, and both IL-10 and AREG can promote angiogenesis. IL-10 is demonstrated to promote the proliferation of CD31^+^ endothelial cells under hyperglycaemic conditions, although the mechanism for AREG in inducing angiogenesis remains unknown.

These findings support the ability of Treg to promote angiogenesis and proliferation of endothelial cells in specific conditions including hypoxia, ischaemia and hyperglycaemia (Figure 3). In addition to their paracrine effect, Treg could also mediate angiogenesis by inhibiting the activities of CD4^+^ Th1 cells 54 or CD8^+^ T cells 56. In mice with normal blood glucose levels, Treg facilitate endothelial cell proliferation and regeneration by sprouting angiogenesis after ischemic injury 54. However, such a regenerative ability of endothelial cells is lost in diabetic mice in which sprouting angiogenesis is negatively regulated by CD4^+^ Th cells 54 and CD8^+^ T cells 56, respectively. Blocking activation of these cells by their respective checkpoint inhibiting antibodies rescues vascular regeneration in diabetic mice, suggesting that Treg that inhibit their activation could also promote angiogenesis in a similar manner. Furthermore, Treg mediated response is often antigen specific. It has been demonstrated that induced angiogenesis by Treg is found only in the ischaemic but not the non-ischemic limb 54. Moreover, the pro-angiogenic effects by Treg may be unique to the tissue type. For instance, lung tissue resident Treg are found to suppress angiogenesis by promoting the apoptosis of murine pulmonary endothelial cells through the DLL4-Notch signalling pathway 57. Therefore, future studies should explore the self-antigen and tissue specificity of Treg in the regulation of angiogenesis, and elucidate a more complete profile of paracrine factors derived from them that might give insights into the development of therapeutics promoting tissue specific angiogenesis.

### Cardiomyocyte repair and regeneration

**Figure 4:**
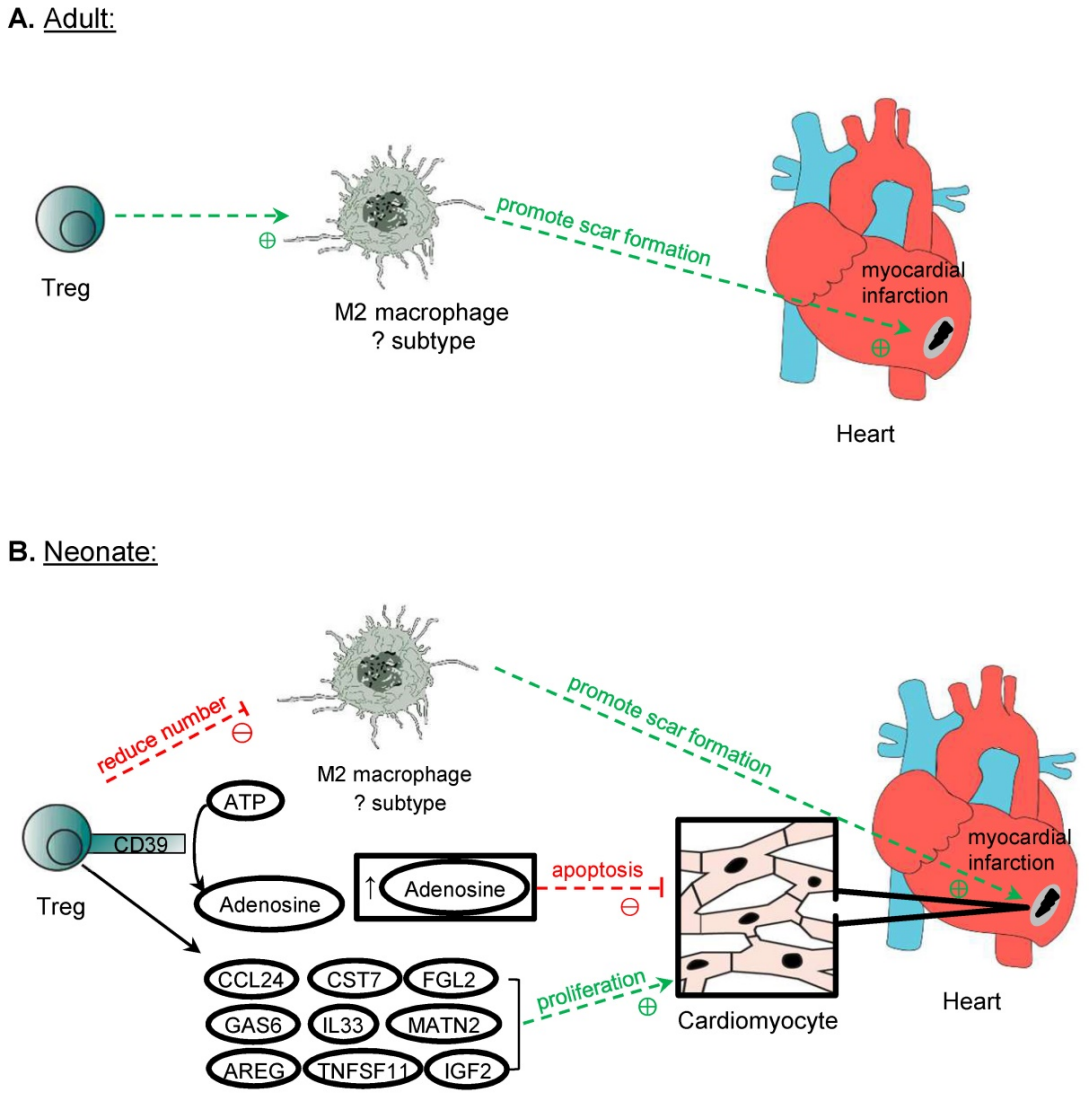
** Treg in cardiomyocyte regeneration.** Treg is crucial in the repair of myocardium post-MI in both adult and neonatal mice. One mechanism proposed is the modulation of M2 macrophages, which promote the formation of scar tissue after MI. However, perhaps due to the different subtypes of M2 macrophages identified in neonatal and adult mice in the myocardium, Treg promotes the expression of M2 cytokines by M2 macrophages in adults but suppresses them in neonatal mice post-MI. Another mechanism of promoting myocardium repair is through the production of adenosine from CD39 expressed on Treg cell surface, and adenosine protects cardiomyocytes from apoptosis by activating RISK pathway. Treg can also directly influence the proliferation of cardiomyocytes in neonates and adults after MI. Even though adult cardiomyocytes do not possess inherent regeneration potential unlike neonatal cardiomyocytes, after MI, Treg promotes the proliferation of cardiomyocytes despite the mechanism remains unknown. Several paracrine factors released by Treg have been identified to promote neonatal cardiomyocytes proliferation, and they include CCL24, GAS7, AREG, CSR7, TNFSF11, IL33, FGL2, MATN2, and IGF2.

In lower vertebrates such as the zebrafish, Treg have been demonstrated to promote adult heart regeneration 58. In mammals, however, the adult heart is notorious in its inability to regenerate as proliferation of adult cardiomyocytes is negligible that cannot account for the recovery of large necrotic areas 59. Unlike the adult heart, the mammalian neonatal heart is still capable of regenerating as cardiomyocytes can still proliferate during the first week of age 60 but the degree of regeneration is dependent on the size of injury 61. Nevertheless, Treg have been demonstrated to facilitate adult heart repair and neonatal heart regeneration after myocardial injuries 62-64, possibly through different mechanisms of action (Figure 4). The ablation of Treg with diphtheria toxin results in left ventricular dilatation and reduced cardiac function in the adult FOXP3^DTR^ mice at 7 days after myocardial infarction (MI) 62. Similarly, the administration of the lytic anti-CD25 antibody that depletes Treg increases lethal outcomes including apical aneurysm and cardiac rupture in the adult mice after MI compared to the untreated control group 63. In addition, the depletion of Treg by the anti-CD25 antibody or ablation of Treg with diphtheria toxin in FOXP3^DTR^ mice results in an increased histological features of cardiac fibrosis and reduced proliferation of cardiomyocytes after injury in the neonatal stage 64. Adoptive transfer of Treg, on the other hand, promotes neonatal heart regeneration by facilitating cardiomyocyte proliferation 64; and improves cardiac function and suppresses adverse cardiac remodelling in the adult mouse heart after injury 65. The administration of the superagonistic anti-CD28 antibody that shows to activate Treg can also lead to reduced cardiac rupture and improved survival 62. Altogether, these reports suggest that Treg offer protection to prevent lethal complications and buffer the deterioration of cardiac function after myocardial injuries through reducing the adverse remodelling of the myocardium in the adult heart, and reducing fibrosis and increasing regeneration of cardiomyocytes in the neonatal heart.

Mechanistically, it has been proposed that Treg facilitate the repair of heart tissue and cardiac function by immunomodulation of macrophages. An *in vitro* study shows that Treg promote the modulation of macrophages to a M2-like phenotype 62. However, in the context of heart recovery, it remains unclear whether the cell fate of macrophages can be polarized in the cardiac tissue after injury; and whether the conversion to M2 macrophages is beneficial to the repair of the myocardium especially in the neonatal heart where fibrosis is less commonly seen than the adult counterpart. In the neonatal mice, an increased level of M2 macrophages is found in the non-regenerating heart when Treg are ablated, whereas a reduced level of M2 is observed in the regenerating heart after adoptive transfer of Treg 64. In the adult mice, there are reduced expression levels of M2 and elevated levels of M1 cytokines in the scar tissue after the ablation of Treg 62. One reason to explain the difference is that the populations of macrophages in the neonatal and adult myocardium could be distinct phenotypically and functionally given the difference in the developmental stages. For instance, 4 distinct populations can be detected in the adult mice distinguished by the expression levels of CCR2, MHCII, LYVE1 and TIMD4 66. The proportions and functions of different subpopulations of cardiac macrophages have altered after MI 67, 68. Furthermore, neonatal heart repair heavily relies on resident macrophages that promote cardiomyocyte proliferation and coronary angiogenesis; whereas adult cardiomyocytes cannot proliferate so the recruited peripherally derived macrophages could operate differently 69. Although M2 macrophages are associated with anti-inflammatory processes and promotion of repair, the excessive action of M2 macrophages may be detrimental as they can contribute to excessive pro-fibrotic function, leading to increased scarring 70. Indeed, both mouse and human pathological specimens of the ischaemic heart show that cardiac fibrosis is associated with an increase in activated macrophages 71; and a recent study also reveals that macrophages contribute collagen to scar formation 72. Nevertheless, the neonatal heart is less fibrotic than the adult heart, the pro-fibrotic M2 macrophages might not be beneficial for neonatal heart regeneration. How Treg regulate the polarization of macrophages during development that may inherently possess different extent of reparative properties remains elusive, and further studies should further stratify the phenotype and function of different populations of macrophages in heart repair.

In addition to the immunomodulation of macrophages, Treg can also suppress the pro-inflammatory response of other activated cells such as effector CD4^+^ and CD8^+^ T cells. Intriguingly, a recent study shows a developmentally distinct role of CD4^+^ T cells in heart repair and regeneration 73. CD4^+^ T cells inhibit heart regeneration in the juvenile mice but promote heart repair in the adult mice 73. Unlike the CD4^+^ T cells, CD8^+^ T cells are found unresponsive to heart injury in the juvenile mice 73. The pro-inflammatory cytokines of Th1 (e.g. TNF-α and IFN-γ) and Th17 (e.g. IL-17A) cells can directly inhibit the proliferation and promote the apoptosis of neonatal cardiomyocytes *in vitro*. Therefore, it is likely that Treg can also facilitate heart regeneration through inhibiting the pro-inflammatory activities of CD4^+^ Th1 and Th17 cells after myocardial injury.

Treg have also been shown to reduce the apoptosis of cardiomyocytes through a CD39 dependent mechanism. *In vivo,* the adoptive transfer of CD39 deficient (CD39^-/-^) Treg fails to reduce the infarct size and cTnT levels after MI compared to the transfer of control Treg 74. It has been previously reported that CD39 can catalyse the production of adenosine 29. Co-culturing neonatal cardiomyocytes with adenosine alone reproduces the reduction of apoptosis seen in cardiomyocytes co-cultured with control Treg, whereas co-culture with the CD39 deficient Treg shows increased apoptosis 74. Furthermore, the adoptive transfer of control Treg leads to increased activation of ERK1/2 and Akt, which belong to the RISK pathway; whereas the same activation is not observed after adoptive transfer of CD39-deficient Treg 74. Therefore, Treg could protect cardiomyocytes from apoptosis through the CD39/adenosine/RISK pathway.

Furthermore, Treg can regulate cardiomyocyte regeneration by directly influencing their proliferation in the neonatal stage. The impaired regenerative capability of adult cardiomyocytes can be attributed not only to their binucleated property leading to cell cycle arrest but also to their developmentally different immune responses after injury. For instance, neonates have less effective Th1 and Th17 cell mediated responses that are poised to a Th2 immunity; and differentiation of Treg from naïve CD4^+^ T cells appears to be a default program 75. Therefore, it is likely that Treg mediated cardiomyocyte regeneration is more apparent in the neonates. The underlying mechanism is suggested to be a paracrine effect, as the culture medium of Treg alone is sufficient to promote neonatal cardiomyocyte proliferation 64. Further analysis of the genes upregulated in Treg after myocardial injury by single-cell RNA-sequencing and by studying the proliferative role of these factors in the neonatal cardiomyocyte cultures allowed the identification of potential candidates that can promote the proliferation of cardiomyocytes 64. These factors include CCL24, GAS6, AREG, CST7, TNFSF11, IL33, FGL2, MATN2, and IGF2, and all of them were found to play a role in stimulating the proliferation of cardiomyocytes 63, 64. These findings might have expanded the repertoire of the reparative capabilities of Treg through the communication with local parenchymal cells such as cardiomyocytes to promote tissue repair and regeneration.

The above evidence supports the notion that Treg participate in adult heart repair and neonatal heart regeneration after injury. How Treg respond to self-antigens after injury has been less studied. It has been reported that Treg of the myocardium can originate from several sources. Adoptive transfer of hCD2-expressing Treg by intraperitoneal injection into the Treg-deficient NOD/SCID mice shows that Treg can be found in the damaged myocardium during the first week after injury, suggesting that Treg can be recruited from the circulation 64. Moreover, Treg are recruited through a CCR5-dependent mechanism to the myocardium after MI 76, and the recruited cardiac antigen-specific CD4^+^ T cells can be converted into Treg with the expression of FoxP3 and factors that promote healing 77. In addition to differentiation from CD4^+^ T cells, proliferation of pre-existing Treg has also been reported. In one study, a small population of Treg is found to reside in a healthy heart of the adult mice, and the proliferation rate of Treg is elevated after injury resulting in an increased overall number of cardiac Treg 78. Nevertheless, future work is required to determine what self-antigens can trigger the recruitment of reparative and regenerative functions of cardiac Treg; what signalling pathway can guide Treg expansion or differentiation from naïve Treg; and whether the reparative property of Treg is inherent to Treg, or exclusive to a particular subtype of Treg such as Foxp3^+^ Treg.

## Clinical implications for treatment of cardiovascular diseases by Treg

Although there is a lack of clinical trial targeting the regenerative role of Treg, murine studies have already shown promising results of using Treg as a therapeutic strategy against cardiovascular diseases. For instance, adoptive transfer of Treg reduces cardiac fibrosis and promotes cardiac regeneration in the neonatal mice after cryoinfarction or apical resection 64. Moreover, direct injection of Treg to the infarct zone of an adult heart after MI showed cell engraftment, leading to reduced infarct size and improved cardiac function for up to 3 months after injury 63. Future research could examine the efficacy of Treg mediated tissue repair in humans because it is clinically feasible to expand or activate Treg. Currently, several clinical trials have been launched to examine the immunosuppressive function of Treg for potential treatment of autoimmune diseases through cytokine therapy, checkpoint blockade or adoptive transfer to enrich the Treg population.

For instance, IL-2 is essential for the growth and stability of Treg that express the IL-2 receptor (CD25). However, a challenge exists as IL-2 does not solely activate Treg but also other effector cells including CD4^+^ T cells, CD8^+^ T cells, or NK cells. To minimize non-specific activation, low-dose IL-2 has been demonstrated to selectively activate and expand Treg 79, 80. Indeed, clinical trials utilizing low dose IL-2 have demonstrated safety and efficacy in the selective activation and expansion of Treg across 11 autoimmune diseases including rheumatoid arthritis, ankylosing spondylitis and systemic lupus erythematosus 81. More recently, IL-2 mimics have been developed but research shows that these mimics contributed to expansion of immunosuppressive, antigen-specific Treg to a lesser extent compared to murine IL-2 82. More work is, therefore, needed to improve the efficiency and efficacy of IL-2 mimics in potential clinical use for Treg expansion. Moreover, targeting the PD-1/PD-L1 pathway can also expand Treg in the context of transplantation 83, 84. Currently, there are already several anti-PD-1 or -PD-L1 antibodies used in the clinics for cancer immunotherapy so it could be in principle safe to test their effects in expanding Treg for tissue repair and regeneration. Furthermore, the CD28 superagonist has been re-investigated recently in humans. At a low dose, CD28 superagonist has been shown to preferentially expand and activate Treg *in vitro*, without the release of pro-inflammatory cytokine release by conventional T cells, which was thought to be the reason behind the fatal cytokine release syndrome experienced by patients in an earlier trial of the same drug 85. Although CD28 superagonist is examined for its efficacy in small groups of patients in phase I trials for psoriasis, rheumatoid arthritis, and systemic lupus erythematosus (NCT01990157, NCT02796053, and NCT02711813, respectively), the results have not been officially published. Therefore, it remains unknown whether low dose CD28 superagonist is a safe and effective therapeutic agent to induce the immunosuppressive effects of Treg in humans.

Furthermore, adoptive transfer of autologous Treg has been examined as potential cell therapy for treatment of autoimmune diseases. For instances, ongoing clinical trials have been using polyclonal 86 or tissue-specific 87 Treg in patients with Type 1 diabetes. It has been reported that tissue-specific Treg, such as those targeting the pancreatic islets, are 50-100 times more effective in the prevention of disease progression of Type 1 diabetes 87. Treg that are reactive to alloantigens have also shown to be more effective than polyclonal Treg in suppressing graft rejection 88, and alloantigen-reactive Treg are currently tested in clinical trial comparing the efficacy to conventional immunosuppressive drugs 87. CAR Treg have also been developed to allow the development of specific Treg for therapy, and preclinical data have demonstrated that these cells are more effective than polyclonal Treg for reducing allograft rejection after transplanted in a humanised mouse model 89. Our current understanding of the regenerative capability of Treg in promoting angiogenesis and cardiomyocyte regeneration is largely based on murine models. An obvious caveat of animal models is that murine Treg might not truly recapitulate the function of human Treg. These clinical trials using autologous Treg in cell therapy could ease the safety concern and provide some confidence for future investigation on the reparative role of human Treg. Taken together, these same approaches of Treg cell therapy might offer novel clinically relevant insights into the expansion and activation of Treg potentially facilitating tissue repair and regeneration in humans.

## Future Perspectives

Treg take up a small proportion of the total immune cell population. It remains unclear how they exert such a large effect on the regulation of tissue repair and regeneration. One possible mechanism is mediated through the orchestration of other abundantly available immune cells. For instance, they can regulate the polarization of macrophages towards a pro-repair M2 phenotype for the regeneration of skeletal muscle 41. Moreover, macrophages can directly contribute collagen to scar formation during zebrafish heart regeneration and adult mouse heart repair 72. Whether Treg regulate macrophage mediated tissue repair and regeneration; and whether they regulate other immune cells in addition to macrophages for these processes require further investigation. In the context of transplantation, Treg are able to induce more Treg from naïve CD4^+^ T cells to acquire immunosuppressive regulatory functions through the process known as infectious tolerance 90. Nevertheless, whether this infectious tolerance exists in the context of tissue repair and regeneration has not been elucidated.

Currently, most studies that evaluated the regenerative role of Treg focus on the conventional CD4^+^CD25^+^FoxP3^+^ Treg population. In fact, as our knowledge of Treg expands, Treg population is found to be heterogeneous. For instance, induction of T_H_1 like-, T_H_2 like-, and T_H_17 like- Treg have been described under certain conditions, such as infection or a pathogenic process 91-94. In the progression of chronic heart failure, Treg are found to be switched to the IFNγ expressing T_H_1 like- phenotype that have reduced immunomodulatory abilities and increased anti-angiogenic and pro-fibrotic functions after the healing phase of MI 78. Moreover, follicular regulatory T cells (T_FR_), which are characterised by CD4^+^CXCR5^high^PD-1^high^Foxp3^+^, can also display suppressive mechanisms through GITR, IL-10, and ICOS, and function in the germinal centre to suppress the action of follicular helper T cells, and inhibit the selection for non-antigen-specific B cells 95. In addition, CD8^+^ Treg are characterized as CD8^+^CD28^-^ and the majority of them also express FoxP3 96. CD8^+^CD28^-^ T cells were able to suppress CD4^+^ T cells by inducing apoptosis, inhibiting T cell activation and IFNγ expression through the release of IL-10, and reducing T cell proliferation via the release of IL-10 and TGF-β 97. Importantly, some T cell subsets have regulatory functions but do not express FoxP3 including the CD8^+^CD122^+^ Treg 98, CD4^+^ Tr1 and Th3 Treg 99, CD4^-^CD8^-^ αβ T cells 100, γδ T cells 100 and even the regulatory innate lymphoid cells 101. Therefore, further work can also focus on the investigation of the potential regenerative role of the heterogeneous Treg population in addition to the conventional CD4^+^CD25^+^FoxP3^+^ cells in experiments related to tissue repair and regeneration.

While a large body of evidence uncovers the emerging role of Treg in promoting tissue repair and regeneration, some reports argue that Treg can also display detrimental function in disease states. For instance, patients with cardiovascular disease often have associated comorbidities such as hypertension and/or diabetes, and Treg has been reported defective in these comorbidities. Analysis of samples from patients with heart failure in one study and coronary artery disease in another study have both showed that the number of Treg of the diseased patients is reduced and Treg are less efficient in suppressing the proliferation and release of pro-inflammatory cytokines by CD4^+^CD25^-^ T cells compared to healthy controls 102, 103. Consistent with these findings from human subjects, a murine model of chronic heart failure has also demonstrated that 90% of cardiac Treg express pro-inflammatory cytokines such as IFNγ and display reduced immunosuppressive ability and anti-angiogenic effect 78. Their negative impact on adverse cardiac remodelling and impaired cardiac function of the failing heart can be abolished by specific ablation of Treg in Foxp3-DTR mice compared to that of the controls 78. Furthermore, an epitope of the atheroprotective apolipoprotein B (ApoB) is found to be recognized by CD4^+^FOXP3^+^ Treg in healthy human subjects that is, however, recognized by Treg coexpressing other CD4 lineage markers in donors with subclinical cardiovascular disease 104. These cells are absent in healthy donors but can also be found in patients with inflammatory disease 104. Therefore, their role in promoting or protecting against atherosclerosis requires future investigations. Altogether, in certain pathological conditions, Treg also exhibit pro-inflammatory properties that could accelerate disease progression and may even operate against tissue repair and regeneration. Future studies characterising the plasticity of Treg function in the presence of cardiovascular comorbidities are needed to better understand their pathogenic role in disease development and progression.
